# Allergy and Parasites

**DOI:** 10.1155/2013/502562

**Published:** 2013-02-12

**Authors:** Fabrizio Bruschi, Maria Ilma Araujo, William Harnett, Elena Pinelli

**Affiliations:** ^1^Department of Translational Research and New Technologies in Medicine and Surgery, University of Pisa, 56126 Pisa, Italy; ^2^Serviço de Imunologia, Universidade Federal da Bahia (UFBA), 40110-160 Salvador, BA, Brazil; ^3^Strathclyde Institute of Pharmacy and Biomedical Sciences, University of Strathclyde, Glasgow G4 0RE, UK; ^4^Centre for Infectious Disease Control Netherlands, National Institute of Public Health and the Environment (RIVM), Bilthoven 3720 BA, The Netherlands

It is estimated that at least one-fifth of the world's population suffers from allergic diseases (atopic asthma and allergic rhinitis are the most common). Different factors contribute to the development of allergies; a predisposing genetic background is needed; however, environmental factors play an important role, among them is the exposure to infectious agents.

Allergy is increasing in the recent decades particularly in Western industrialized countries, and according to the so-called *hygiene hypothesis* this might be ascribable to the lower exposure to infectious agents, including parasitic helminths. The reasons for such a situation are several: small family size, stability of intestinal microflora, affluent urban homes, high use of antibiotics, good sanitation which means low oral-fecal pathogen burden, and finally low or absent helminth infections. On the other hand, the low prevalence of atopy in developing countries is generally associated with high exposure to parasites in these regions.

The hygiene hypothesis has been revisited pointing out, for example, the role of IL-10-producing Treg cells, which are induced during chronic infections and play an essential role in downregulating allergic responses.

To verify the above hypotheses, a number of publications have appeared, studying the effects of parasitic infections or of molecules derived from parasites on the well-established experimental models of allergy, along with epidemiological studies, which have tried to correlate these conditions.

This special issue on allergy and parasites is composed of papers, which address this topic.

A review by N. Rujeni et al. focuses on the relationship between helminth parasites and allergic reactivity, taking into account the new concept to explain why Th2 immune responses have evolved. The Th2 response has been associated with host resistance to helminth infections and generally considered a harmfully allergic response. The review discusses these concepts and also the mechanisms underlying the interface between allergy and helminth infections and the evolutionary processes of the immune response associated with these conditions.

In the paper by D. Ball et al., the influence of ES-62, a glycoprotein produced by *Acanthocheilonema viteae*, on the activity of mast cells of different sources (peritoneal, connective tissue, and bone marrow derived) was assayed, comparing surface phenotype, degranulation responses to LPS and Fc*ε*R1 cross-linking, and cytokine production in response to those stimuli. The authors show the activation pathways in mouse mast cells that may be altered by ES-62. These results will advance our understanding of the immunomodulatory activity directed against the allergy effector cells by this parasite-derived glycoprotein.

C. Aranzamendi et al. observe that the association between allergy and helminth infections is not always the same. These authors review the literature on some of the cell types that play an essential role in helminth-induced immunoregulation and the consequences for inflammatory diseases. In addition, epidemiological as well as experimental studies indicating the contrasting effects of *Toxocara* and *Trichinella *infection on allergic asthma are discussed. According to the authors, this diversity might depend on the helminth species, whether it is a chronic or acute infection, the different role played by the host in the various infections (natural or occasional), as well as on the different life cycles in which the parasite may affect the lung (in toxocarsis) or not (in trichinellosis), and on parasite burden.

Several epidemiological studies have consistently shown an inverse association between clinical asthma and *Schistosoma* infections. M. C. F. Almeida et al. carried out an elegant intervention study using the appropriate method to assess whether helminth infections are associated with asthma symptoms. The method used was a prospective, double-blinded, placebo-controlled study evaluating the influence of antihelminthic treatment on asthma severity, carried out in an endemic area for schistosomiasis. Findings on worsening of asthma severity after repeated antihelminthic treatments support the hypothesis on the protective effect of helminth infections in inflammatory diseases. Additional studies, such as the one described by Almeida et al. which would include a larger number of participants and continue for a longer period of time, are necessary in order to elucidate further the association between different helminth infections and inflammatory diseases.

In the paper by L. S. Cardoso and colleagues, risk factors for asthma were investigated in an area of helminth transmission in Bahia, Brazil, by the use of questionnaires. The effect of *Schistosoma mansoni* and also various gastrointestinal nematodes was investigated. Although the authors could not conclude that schistosomes offered a protective effect, the results indicated that *Ascaris lumbricoides* infection was negatively associated with asthma. This paper may thus be consistent with the idea referred to earlier that parasitic worm species vary in their immunomodulatory effects, but it should also be noted (as outlined above) that other studies show schistosome infection to be associated with protection against allergy.

In conclusion, the editors hope to have stimulated, with this special issue, the interest on the relationship between allergy and parasitic infections; envisaging new research work is needed not only to clarify many aspects which remain uncertain, but also to open the way to possible new strategies to control allergic diseases.

*Fabrizio Bruschi* *Fabrizio Bruschi* *Maria Ilma Araujo* *Maria Ilma Araujo* *William Harnett* *William Harnett* *Elena Pinelli* *Elena Pinelli*

